# Artificial intelligence‐driven consensus gene signatures for improving bladder cancer clinical outcomes identified by multi‐center integration analysis

**DOI:** 10.1002/1878-0261.13313

**Published:** 2022-09-22

**Authors:** Hui Xu, Zaoqu Liu, Siyuan Weng, Qin Dang, Xiaoyong Ge, Yuyuan Zhang, Yuqing Ren, Zhe Xing, Shuang Chen, Yifang Zhou, Jianzhuang Ren, Xinwei Han

**Affiliations:** ^1^ Department of Interventional Radiology The First Affiliated Hospital of Zhengzhou University China; ^2^ Interventional Institute of Zhengzhou University China; ^3^ Interventional Treatment and Clinical Research Center of Henan Province Zhengzhou China; ^4^ Department of Colorectal Surgery The First Affiliated Hospital of Zhengzhou University China; ^5^ Department of Respiratory and Critical Care Medicine The First Affiliated Hospital of Zhengzhou University China; ^6^ Department of Neurosurgery The Fifth Affiliated Hospital of Zhengzhou University China; ^7^ The First Affiliated Hospital of Zhengzhou University China

**Keywords:** biomarker, bladder cancer, immunotherapy, multi‐omics, prognosis

## Abstract

To accurately predict the prognosis and further improve the clinical outcomes of bladder cancer (BLCA), we leveraged large‐scale data to develop and validate a robust signature consisting of small gene sets. Ten machine‐learning algorithms were enrolled and subsequently transformed into 76 combinations, which were further performed on eight independent cohorts (*n* = 1218). We ultimately determined a consensus artificial intelligence‐derived gene signature (AIGS) with the best performance among 76 model types. In this model, patients with high AIGS showed a higher risk of mortality, recurrence, and disease progression. AIGS is not only independent of traditional clinical traits [(e.g., American Joint Committee on Cancer (AJCC) stage)] and molecular features (e.g., *TP53* mutation) but also demonstrated superior performance to these variables. Comparisons with 58 published signatures also indicated that AIGS possessed the best performance. Additionally, the combination of AIGS and AJCC stage could achieve better performance. Patients with low AIGS scores were sensitive to immunotherapy, whereas patients with high AIGS scores might benefit from seven potential therapeutics: BRD‐K45681478, 1S,3R‐RSL‐3, RITA, U‐0126, temsirolimus, MRS‐1220, and LY2784544. Additionally, some mutations (*TP53* and *RB1*), copy number variations (7p11.2), and a methylation‐driven target were characterized by AIGS‐related multi‐omics alterations. Overall, AIGS provides an attractive platform to optimize decision‐making and surveillance protocol for individual BLCA patients.

## Graphical abstract

Based on multiple bioinformatics and machine‐learning algorithms, we developed a robust and powerful consensus artificial intelligence gene signature (AIGS) that can accurately predict the prognosis, recurrence, and immune response for bladder cancer. In addition, AIGS is also a promising biomarker for predicting chemotherapy response, and the identification of potential compounds demonstrates dramatic implications of precise treatment for high‐risk patients.

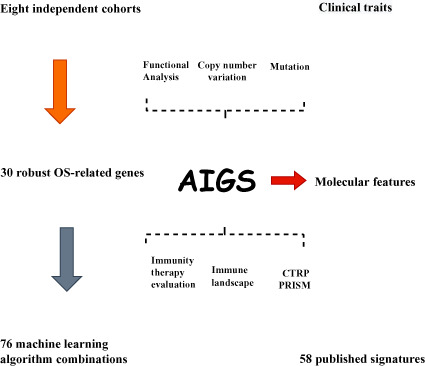

**Abbreviations**
AIGS: artificial intelligence‐derived gene signature
AJCC: American Joint Committee on Cancer
AMP: amplification
BH: Benjamini–Hochberg
BLCA: bladder cancer
CCLs: cancer cell lines
CNV: copy number variation
CTRP: cancer therapeutics response portal
Enet: elastic network
FGG: fraction of genomes gained
FGL: fraction of genomes lost
FMGs: frequently mutated genes.
GBM: generalized boosted regression modelling
GSEA: gene set enrichment analysis
GSVA: gene set variation analysis
Homdel: homozygous deletion
HR: hazard ratio
ICI: immune checkpoint inhibitor
IPS: immunophenoscore
k‐NN: K‐nearest neighbour
MDGs: methylation‐driven genes
OS: overall survival
PFS: progression‐free survival
plsRcox: partial least squares regression for cox
RFS: recurrence‐free survival
RORGs: robust OS‐related genes
RSF: random survival forest
SubMap: subclass mapping
SuperPC: supervised principal components
survival‐SVM: survival support vector machine
TCGA: the cancer genome atlas
Th: T helper
TIDE: tumour immune dysfunction and exclusion
TMB: tumour mutation burden

## 1 Introduction

As one of the most common malignancies globally, over 430 000 patients were diagnosed with bladder cancer (BLCA) in 2020 [1]. Due to the rapid disease progression and undertreatment, BLCA patients performed high mortality, recurrence, and treatment failure rates [2, 3]. Hence, the early identification and intervention of high‐risk BLCA patients exhibit dramatic significance. As a recognized authoritative grading system, the American Joint Committee on Cancer (AJCC) stage system provides a common platform for evaluating the prognostic risk and treatment measures in clinical management. However, different clinical outcomes of the same stage patients and the lack of molecular biological characteristics indicate the limitations of the AJCC classification system [4, 5], which might lead to potential over‐or under‐treatment. Over the past decades, immunotherapy has exhibited a great sensation due to the dramatic benefits of solid cancer treatment, including BLCA [6, 7]. Immune checkpoint inhibitor (ICI) can promote the immune system to recognize and suppress basic molecular targets of tumour cells such as PD‐1, CTLA‐4, and PD‐L1. In the US, PD‐L1 targeting drugs like atezolizumab and pembrolizumab have been approved as first‐line therapy in patients with platinum‐ineligible PD‐L1+ BLCA [8]. Apart from this, there are other molecular classification tools to stratify patients, such as CTLA‐4, PD‐1, and tumour mutation burden (TMB) [9]. Unfortunately, these classification systems do not perfectly predict response to ICI therapy and just a small proportion of BLCA patients can benefit from them [9]. Given the enormous cost and serious adverse effects of immunotherapy, exploring a novel biomarker for effective immunotherapy management in BLCA is also warranted.

As is known to all, BLCA is a comprehensive tumour with inter‐ and intra‐tumour heterogeneity [10]. Ideal biomarkers should possess homogeneous expression within and between tumour tissues to behave stably accord all patients. Thus, multigene panels are possible to be an effective approach to address inter‐ and intra‐cancer heterogeneity. With the rapid development of bioinformatics and computer technology, a large number of gene prognosis signatures have been reported [11, 12]. Models integrated through multiple gene profiles, including messenger RNA, microRNA, and long non‐coding RNA, were constructed and validated as promising biomarkers for BLCA [13, 14, 15]. However, considering the underutilization of data information, inappropriate machine‐learning methods, and lack of rigorous validation in different cohorts and clinical trials, multigene signatures demonstrate dramatic limitations in clinical application [16, 17].

To fill these gaps, we comprehensively investigated the clinical value of BLCA gene expression profiles, and a consensus artificial intelligence‐derived gene signature (AIGS) from the combination of 76 machine‐learning algorithms was systematically developed. The predictive value of AIGS for overall survival (OS), recurrence‐free survival (RFS), progression‐free survival (PFS), immunotherapy, and chemotherapy was tested in 1351 BLCA patients from 11 independent cohorts. After comparing our signature with 58 published models, traditional clinical traits, and molecular features, the clinical translation value and robust performance of AIGS were further validated. Additionally, a methylation‐driven target and some AIGS‐related mutations (*TP53* and *RB1*) and copy number variations (CNVs; *7p11.2*) were obtained based on multi‐omics data analysis, and seven potential therapeutic drugs for high‐risk patients were also identified in our study. Overall, our work provides an attractive platform for recognizing high‐risk patients and optimizing precision treatment for BLCA.

## 2 Materials and methods

### 2.1 Data collection and processing

**Fig. 1:**
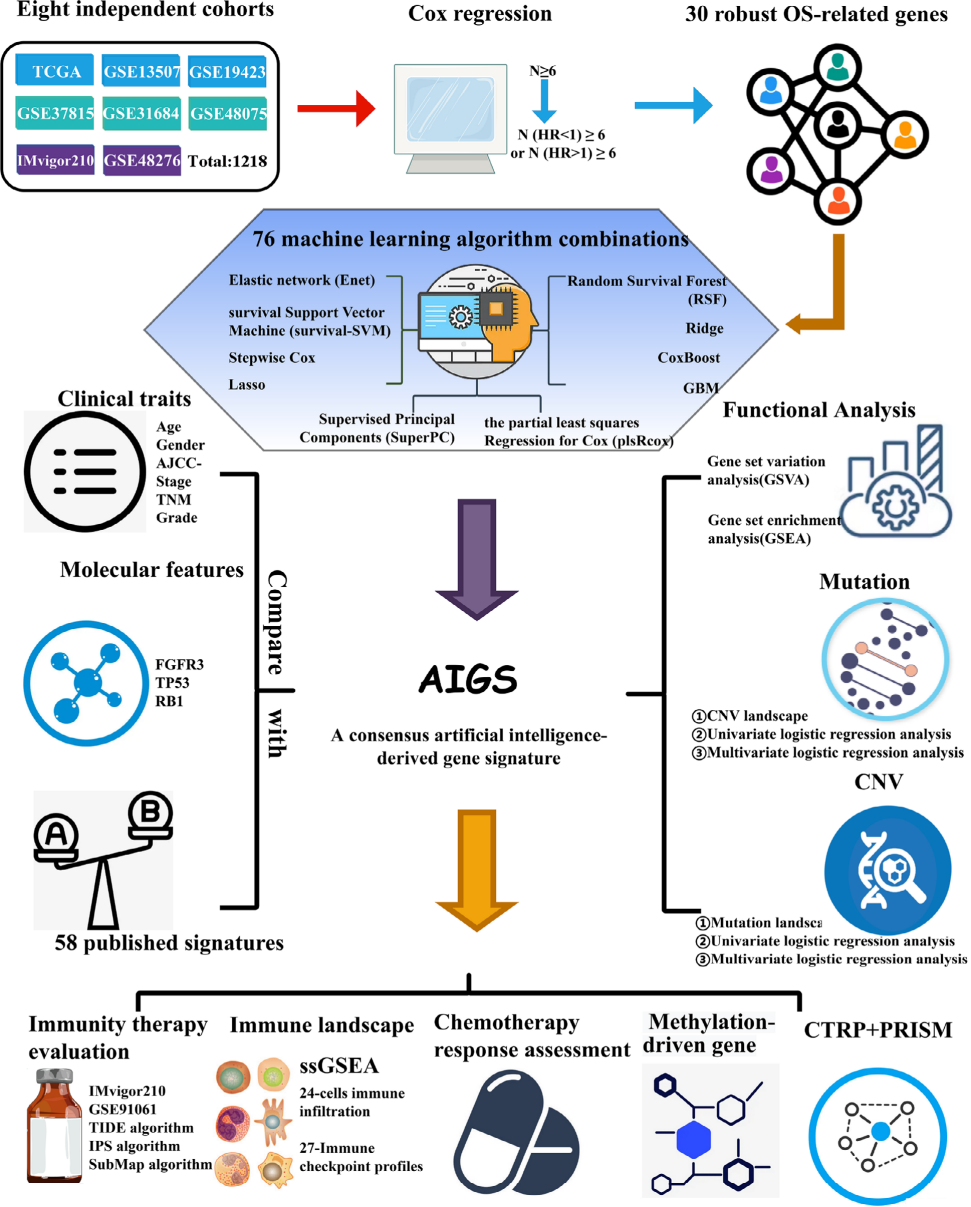
The flow chart of this study. CNV, copy number variation; CTRP, cancer therapeutics response portal datasets; OS, overall survival; ssGSEA, single‐sample gene set enrichment analysis.

The flow chart of our research is illustrated in Fig. 1. A total of 10 independent datasets (*n* = 1003) were retrospectively retrieved from Gene Expression Omnibus and The Cancer Genome Atlas (TCGA) database, respectively. The IMvigor210 cohort (*n* = 348) was enrolled from the published research [18]. Samples were screened according to the following conditions: (a) primary cancer tissues; (b) no preoperative radiotherapy or chemotherapy received; (c) survival information was available; and (d) RNA expression data were available. See Table S1 for the detailed baseline information.

Among the 11 cohorts, eight cohorts (TCGA‐BLCA, GSE13507, GSE19423, GSE31684, GSE37815, GSE48075, GSE48276, and IMvigor210) with complete OS information were employed to construct and validate our AIGS. Two cohorts (GSE31684 and GSE154261) contained RFS, and PFS information was utilized to investigate the predictive performance of AIGS for RFS and PFS, respectively. Two immunotherapy cohorts (including IMvigor210 and GSE91061) and one chemotherapy cohort (GSE52219) were performed to assess the value of AIGS in predicting immunotherapy and chemotherapy responses for BLCA. Of note, the FPKM normalized data from TCGA were further converted into log2 (TPM + 1), and 14 843 intersection genes of these cohorts were obtained for the subsequent analysis.

### 2.2 Generation of AIGS

As previously reported by Liu et al. [19, 20], gene expression profiles were converted into z‐score across all datasets to enhance comparability between different cohorts, which in turn constructed a consensus artificial intelligence‐derived signature. Cohorts with OS information including TCGA‐BLCA, GSE13507, GSE19423, GSE31684, GSE37815, GSE48075, GSE48276, and IMvigor210 were utilized to develop AIGS in as following steps:

1. 14 843 intersection genes of these eight OS cohorts were utilized to perform univariate Cox analysis. Considering the small sample of some cohorts and the strict multiple testing correction that might filter out potential genes associated with OS, genes with both an unadjusted *P* < 0.2 and the same hazard ratio (HR) direction for more than six cohorts were considered as robust OS‐related genes (RORGs).
2. To develop a consensus AIGS with high accuracy and stability performance, we integrated 10 machine‐learning algorithms including random survival forest (RSF), elastic network (Enet), Lasso, Ridge, stepwise Cox, CoxBoost, partial least squares regression for Cox (plsRcox), supervised principal components (SuperPC), generalized boosted regression modelling (GBM), and survival support vector machine (survival‐SVM). A few algorithms possessed the ability of feature selection, such as Lasso, stepwise Cox, CoxBoost, and RSF. Thus, we combined these algorithms to generate a consensus model. In total, 76 algorithm combinations were conducted on RORGs to fit prediction models based on 10‐fold cross‐validation. The initial signature discovery was performed in TCGA‐BLCA. The RSF model was implemented via the *randomForestSRC* package. RSF had two parameters *ntree* and *mtry*, where *ntree* represented the number of trees in the forest and *mtry* was the number of randomly selected variables for splitting at each node. We used a grid search on ntree and mtry using 10‐fold cross‐validation. All the pairs of (*ntree*, *mtry*) were formed, and the one with the best C‐index value was identified as the optimized parameter. The Enet, Lasso, and Ridge were implemented via the *glmnet* package. The regularization parameter, lambda, was determined by 10‐fold cross‐validation, whereas the L1‐L2 trade‐off parameter, α, was set to 0–1 (interval = 0.1). The stepwise Cox model was implemented via s*urvival* package. A stepwise algorithm using the AIC (Akaike information criterion) was applied. The CoxBoost model was implemented via *CoxBoost* package, which is used to fit a Cox proportional hazards model by componentwise likelihood‐based boosting. For the CoxBoost model, we used 10‐fold cross‐validation routine *optimCoxBoostPenalty* function to first determine the optimal penalty (amount of shrinkage). Once this parameter was determined, the other tuning parameter of the algorithm, namely, the number of boosting steps to perform, was selected via the function *cv.CoxBoost*. The dimension of the selected multivariate Cox model was finally set by the principal routine CoxBoost. The plsRcox model was implemented via *plsRcox* package. The *cv.plsRcox* function was used to determine the number of components requested, and the *plsRcox* function was applied to fit a partial least squares regression generalized linear model. The SuperPC model was implemented via *superpc* package and is a generalization of principal component analysis, which generates a linear combination of the features or variables of interest that capture the directions of largest variation in a dataset. The superpc.cv function used the 10‐fold cross‐validation to estimate the optimal feature threshold in superpc. To avoid problems with fitting Cox models to small validation datasets, it uses the ‘pre‐validation’ approach. The GBM was implemented via the *gbm* package. Using the 10‐fold cross‐validation, the *cv.gbm* function selected index for number trees with minimum cross‐validation error. The *gbm* function was used to fit the generalized boosted regression model. The survival‐SVM model was implemented via *survivalsvm* package. The regression approach takes censoring into account when formulating the inequality constraints of the support vector problem.
3. All these 76 algorithms were applied in the other seven OS cohorts. The C‐index across all validation cohorts was calculated for each signature, and signature displayed the highest average C‐indices was considered the optimal one.

### 2.3 Acquisition of published signatures

As illustrated in Table S2, a total of 58 published signatures were comprehensively enrolled. Notably, miRNA signatures were not included in the collection given the lack of miRNA information in our cohorts. Afterwards, univariate Cox analyses were employed, and the C‐indices for each signature across all cohorts were calculated.

### 2.4 Gene set enrichment analysis (GSEA)

Pearson's correlation was utilized to evaluate the correlation between AIGS scores and genes. All genes were arranged in descending order of correlation coefficient, and GSEA was performed by *clusterProfiler* r package to recognize remarkably enriched terms associated with the GO and KEGG pathways, subsequently [21].

### 2.5 Gene set variation analysis (GSVA)

The Hallmark gene set was obtained from the molecular signatures database. According to the median AIGS score, patients were divided into two groups. To reduce the overlap and redundancy of pathways, gene set associated with a pathway was screened to contain unique genes, and genes related to multiple pathways were also removed [22]. The *limma* package was employed to recognize the remarkably altered pathways between the high‐ and low‐AIGS groups, and the pathway with ¦*t*¦ > 1 was regarded as significant.

### 2.6 Comprehensive analyses based on immune cell infiltration and immune checkpoints

The single‐sample Gene Set Enrichment Analysis was performed to assess the infiltration abundance of 24 immune cells in tumour immune microenvironment via *GSVA* package in the IMvigor210 cohort. Gene set of 24 immune cell and 27 immune checkpoints including the member of the TNF superfamily, B7‐CD28 family, and other molecules were retrieved from the published research [23, 24, 25, 26, 27]. Afterwards, the correlations among the AIGS scores, immune infiltration, and immune checkpoints were investigated.

### 2.7 Immunotherapeutic response prediction

The tumour immune dysfunction and exclusion (TIDE), immunophenoscore (IPS), and subclass mapping (SubMap) algorithms were employed to predict the responses to immunotherapy from multiple perspectives [28, 29, 30]. TIDE is an algorithm that evaluates immune evasion by integrating the expression characteristics of T‐cell exclusion and T‐cell dysfunction. IPS was proved to be a superior predictor to identify determinants of immunogenicity and characterize the intratumoral immunologic landscape [31], and SubMap was utilized to derive the degree of commonality between high‐ and low‐AIGS groups. Notably, the adjusted *P*‐value was employed to assess the similarity, and a lower adjusted *P*‐value represents a higher similarity.

### 2.8 The mutation landscape of BLCA

The somatic mutation (VarScan2 variant aggregation and masking) and HumanMethylation450 array were downloaded from the TCGA GDC website, and TMB was obtained by calculating the count of non‐silent somatic mutation in every patient. To investigate the differences in genomic mutations between high‐ and low‐AIGS patients, the mutation waterfall plot of the top 30 genes with the highest mutation number was visualized via the *maftools* and *ComplexHeatmap* r packages. Afterwards, univariate and multivariate logistic regression analyses were employed to evaluate and verify the relationship between 30 genes mutation status and AIGS. It is worth noting that apart from age, gender, and stage, TMB was also included in the multivariate logistic regression to ensure that AIGS was an independent factor for these mutated genes.

### 2.9 CNV for BLCA patients

CNV data processed by the Genomic Identification of Significant Targets in Cancer 2.0 algorithm were retrieved from FireBrowse (http://firebrowse.org/) [32]. The *ComplexHeatmap* package was performed to visualize the CNV waterfall chart of the top 15 amplification (AMP) and homozygous deletion (Homdel) chromosome fragments. To investigate the proportion of genomic alterations, the fraction of genomic alterations, fraction of genomes gained (FGG), and fraction of genomes lost (FGL) were also calculated. Besides, the Wilcox test, univariate and multivariate logistic analyses were employed to assess the correlation between 30 CNV fragments and AIGS. Similarly, FGG and FGL were contained in the multivariate logistic regression analysis to confirm that AIGS was an independent factor of AMP and Homdel fragments, respectively.

### 2.10 Identification of methylation‐driven genes (MDGs)

The HumanMethylation450 array for BLCA was retrieved from TCGA. Referring to the description of Liu et al. [33, 34], the global methylation level was evaluated through averaged beta values of the specific probes. The *MethylMix* r package was used to integrate methylation and mRNA data for correlation analysis. Genes with significantly negatively correlated with expression were defined as MDGs. The relationship between MDGs and AIGS was explored subsequently.

### 2.11 Estimation of drug response and potential therapeutic agents

Drug sensitivity information of cancer cell lines (CCLs) was collected from prism and the Cancer Therapeutics Response Portal datasets (CTRP). Sensitivity data for over 481 compounds can be retrieved in CTRP, and sensitivity data for 1448 compounds in over 482 CCLs are available in prism. Both datasets provide area under the dose–response curve (AUC) value as a measure of drug sensitivity, with lower AUC values indicating higher drug response. K‐nearest neighbour (k‐NN) imputation was employed to impute the missing AUC values, and before imputation, compounds with less than 20% missing values were screened out.

### 2.12 Statistical analysis

The relationship of two variables was obtained by Pearson's correlation. The *Survival* package was utilized to perform Kaplan–Meier survival analysis, and the different significance was determined by the log‐rank test. The ROC curves were plotted by *pROC* package. Differences in AIGS between the high and low groups were compared by independent samples *t*‐test or Wilcoxon rank‐sum test. All statistical *P* values were two‐sided, and *P* < 0.05 was defined as statistically significant. Adjust *P*‐value was employed using Benjamini–Hochberg (BH) multiple test correction. All data processing and plotting were finished in r 4.1.2 software.

## 3 Results

### 3.1 Integrative construction of AIGS

**Fig. 2:**
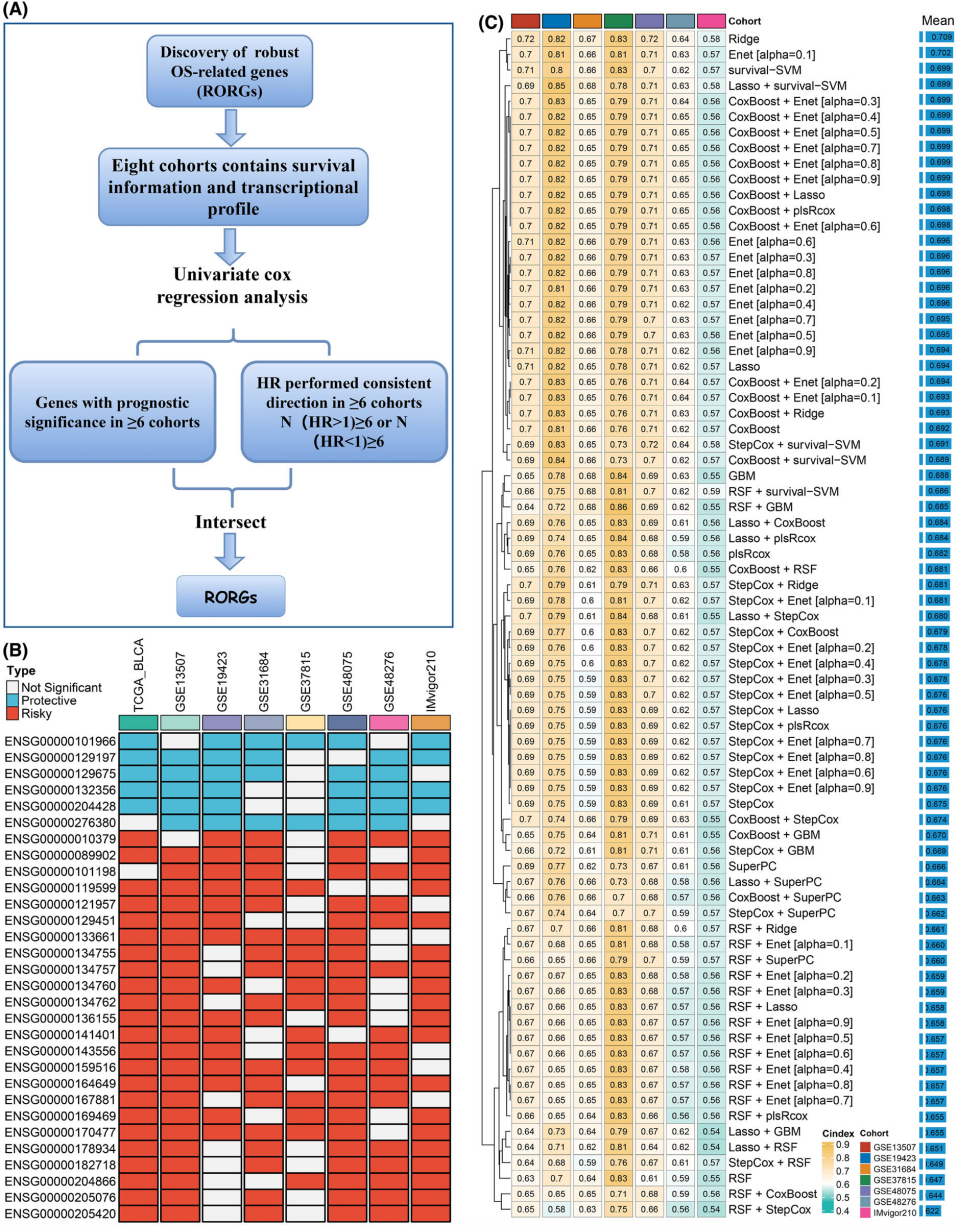
Generation of the artificial intelligence‐derived gene signature. (A) Discovery of robust OS‐related genes (RORGs). (B) 30 robust OS‐related genes were identified in eight cohorts (*n* = 1218). (C) The C‐indices of 76 machine‐learning algorithms in seven validation cohorts (*n* = 818).

Based on the screening criteria in Fig. 2A, a total of 30 RORGs were obtained (Fig. 2B). The expression profiles of these 30 RORGs were utilized to construct the consensus signature subsequently. According to the 10‐fold cross‐validation framework, 76 kinds of prediction signatures were developed in TCGA‐BLCA cohort. The C‐index was employed to assess the predictive ability of these 76 models. Additionally, to verify whether the models performed consistently in the different cohorts, the C‐index of each model was calculated across the remaining seven validation cohorts. As Fig. 2C displayed, Ridge was confirmed to be the optimal signature with the highest average C‐index (0.709). Based on the expression and weighted regression coefficients of the 30 RORGs, a risk score for each patient was further calculated. For the annotation information of these 30 RORGs, please refer to Table S3.

### 3.2 Independent prognostic value of AIGS

**Fig. 3:**
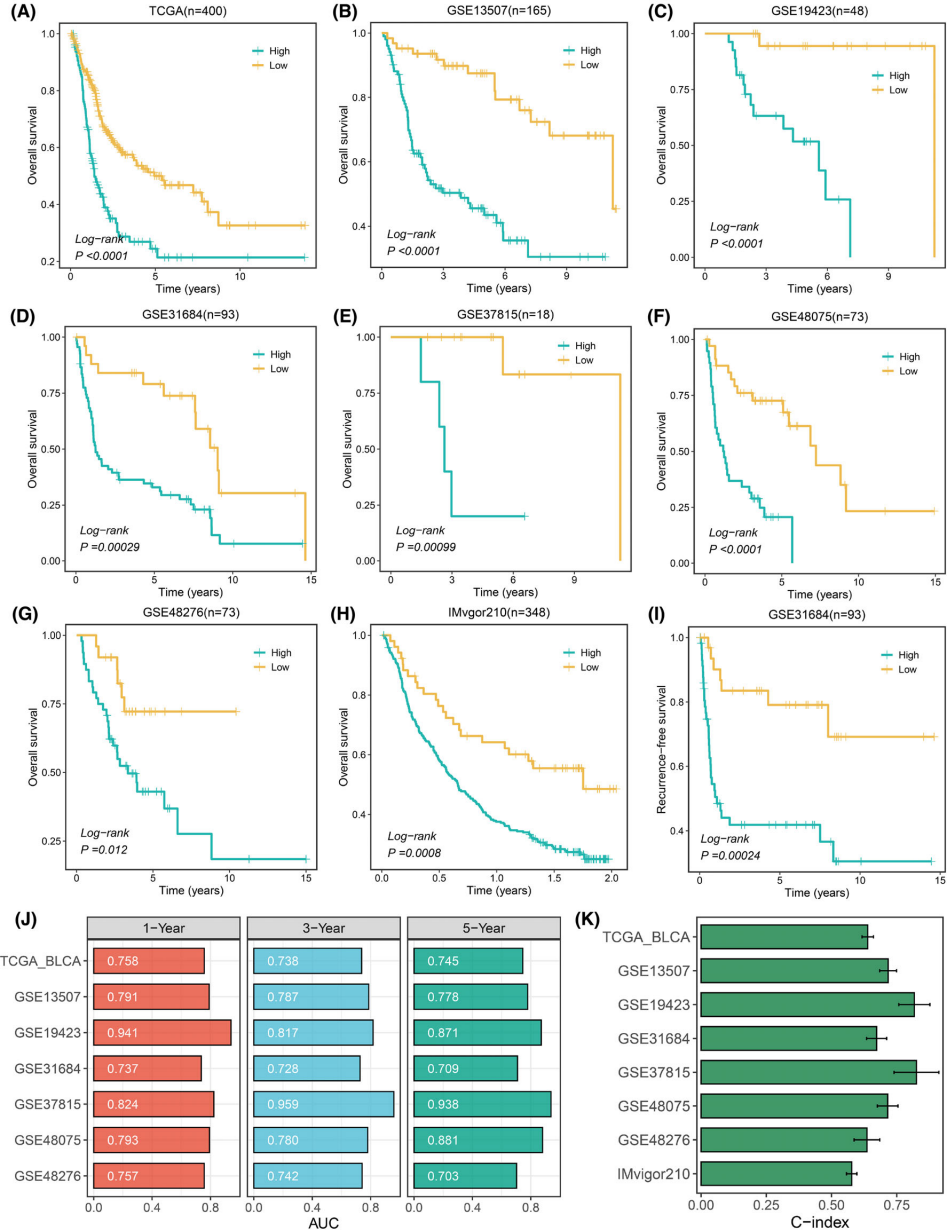
Survival analysis and performance evaluation of artificial intelligence‐derived gene signature (AIGS). (A–H) Kaplan–Meier survival analysis between the high and low AIGS groups across eight OS cohorts. (I) Kaplan–Meier survival analysis between the high and low AIGS groups in GSE31684 (*n* = 93). (J) Time‐dependent ROC analysis for predicting OS at 1, 3, and 5 years in TCGA‐BLCA (*n* = 400), GSE13507 (*n* = 165), GSE19423 (*n* = 48), GSE31684 (*n* = 93), GSE37815 (*n* = 18), GSE48075 (*n* = 73), and GSE48276 (*n* = 73). (K) The C‐indexes of AIGS in TCGA‐BLCA, GSE13507, GSE19423, GSE31684, GSE37815, GSE48075, GSE48276, and IMvigor210 (*n* = 348); the error bars indicate 95% confidence interval (CI).

Kaplan–Meier survival analysis exhibited that the mortality rate in the high‐risk group was significantly higher than the low‐risk group in the training cohort (TCGA‐BLCA, *n* = 400, *P* < 0.0001) and other seven validation cohorts GSE13507 (*n* = 165, *P* < 0.0001), GSE19423 (*n* = 48, *P* < 0.0001), GSE31684 (*n* = 93, *P* = 0.00029), GSE37815 (*n* = 18, *P* = 0.00099), GSE48075 (*n* = 73, *P* < 0.0001), GSE48276 (*n* = 73, *P* = 0.012), and IMvigor210 (*n* = 348, *P* = 0.0008; Fig. 3A–H). Similarly, comparisons of RFS and PFS suggested that the relapse rate in the high‐risk group was significantly higher in GSE31684 (*n* = 93, *P* = 0.00024), and the progress rate in the high‐risk group was dramatically higher in GSE154261 (*n* = 71, *P* = 0.0091; Figs 3I and S1A).

Multivariate Cox analysis was employed to investigate whether the prognostic performance of AIGS was independent after adjusting for clinical factors and molecular features like age, gender, stage, T, N, M, grade, intravesical therapy, systemic chemotherapy, smoking, BCG treatment, platinum therapy, neoadjuvant chemotherapy, FGFR3, p53, RAS, and RB1 mutations. As shown in Tables [Link], [Link], AIGS displayed statistically significant for OS, RFS, and PFS across all cohorts after adjusting these features, suggesting that it was an independent risk factor in BLCA.

### 3.3 Stable performance of AIGS

As displayed in Fig. 3J, AIGS performed superior performance in TCGA with time‐dependent AUCs 0.758/0.738/0.745 at 1, 3, and 5 years, respectively. Similar results were also obtained across the validation cohorts GSE13507 (0.791/0.787/0.778), GSE19423 (0.941/0.817/0.871), GSE31684 (0.737/0.728/0.709), GSE37815 (0.824/0.959/0.938), GSE48075 (0.793/0.780/0.881), and GSE48276 (0.757/0.742/0.703). Notably, the AUCs of the IMvigor210 cohort were 0.712/0.733/0.794 at 0.5, 1, and 2 years due to the max OS time of less than 3 years (Fig. S1B). The C‐index (95% confidence interval) was 0.639 (0.617–0.660), 0.717 (0.684–0.749), 0.817 (0.758–0.877), 0.673 (0.634–0.711), 0.825 (0.740–0.911), 0.715 (0.676–0.755), 0.636 (0.587–0.685), and 0.577 (0.557–0.597) in eight cohorts, respectively (Fig. 3K). All of the above results demonstrated that AIGS possessed superior stability and extrapolation across multiple independent cohorts.

**Fig. 4:**
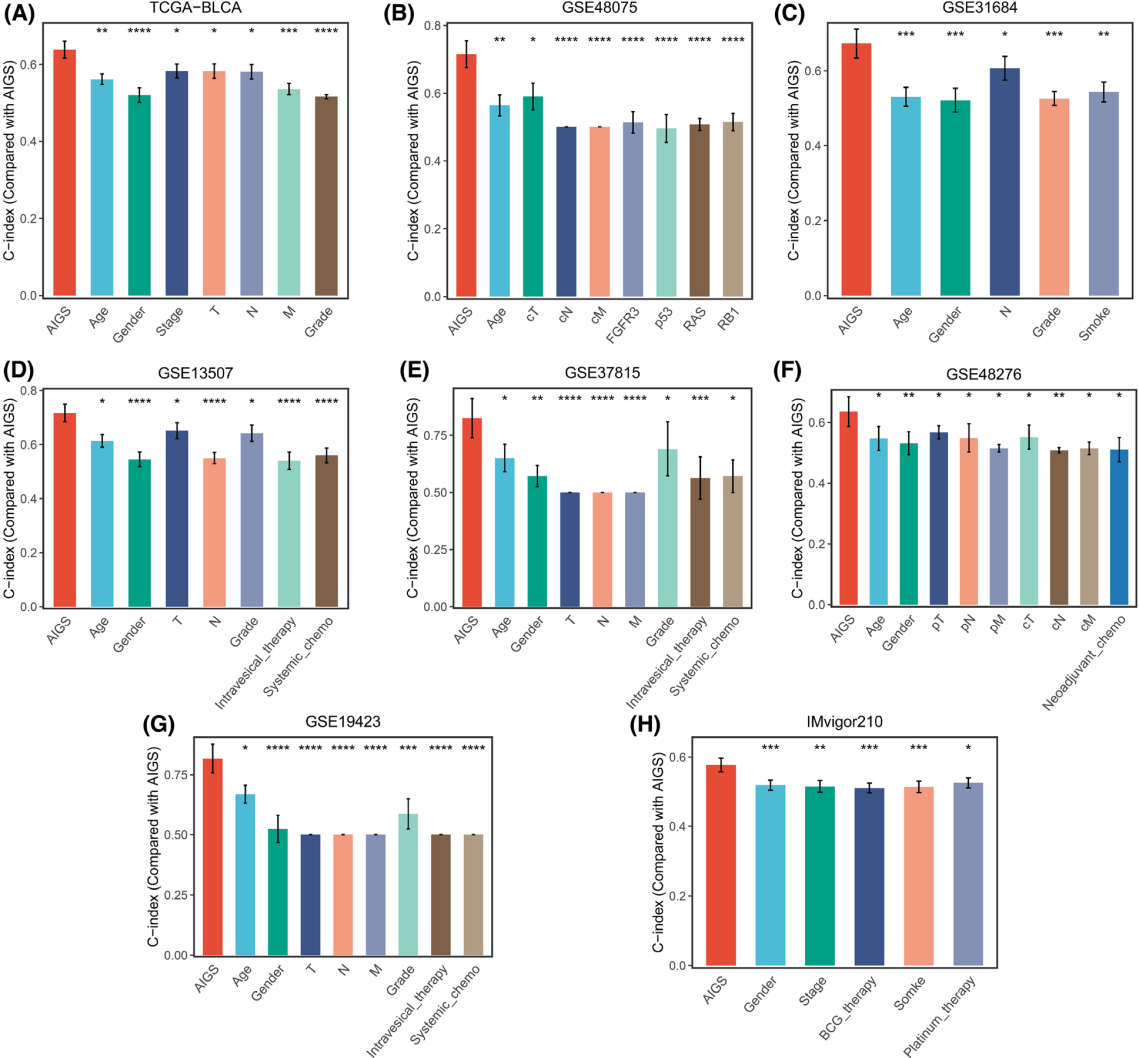
The performance of AIGS was compared with common clinical and molecular variables in predicting prognosis across all training and validation cohorts. The error bars indicate 95% confidence interval (CI). TCGA‐BLCA (*n* = 400), GSE13507 (*n* = 165), GSE19423 (*n* = 48), GSE31684 (*n* = 93), GSE37815 (*n* = 18), GSE48075 (*n* = 73), GSE48276 (*n* = 73), and IMvigor210 (*n* = 348). Z‐score test: *P < 0.05, **P < 0.01, ***P < 0.001, ****P < 0.0001. cM, Clinical M stage; cN, Clinical N stage; cT, Clinical T stage; M, M stage; N, N stage; T, T stage.

Over the past few decades, some clinical traits and molecular features played a fundamental role in prognosis risk assessment and clinical decision optimization. Therefore, we compared the prediction accuracy of AIGS with these variables. As illustrated in Fig. 4A–H, AIGS exhibited dramatically higher accuracy than these features, including age, stage, gender, T, N, M, grade, intravesical therapy, systemic chemotherapy, smoking, BCG treatment, platinum therapy, neoadjuvant chemotherapy, *FGFR3*, *p53*, *RAS*, and *RB1* mutations, revealing that our AIGS potential to be a promising tool in evaluating the prognosis risk of BLCA.

### 3.4 Comparisons between AIGS and 58 published signatures

**Fig. 5:**
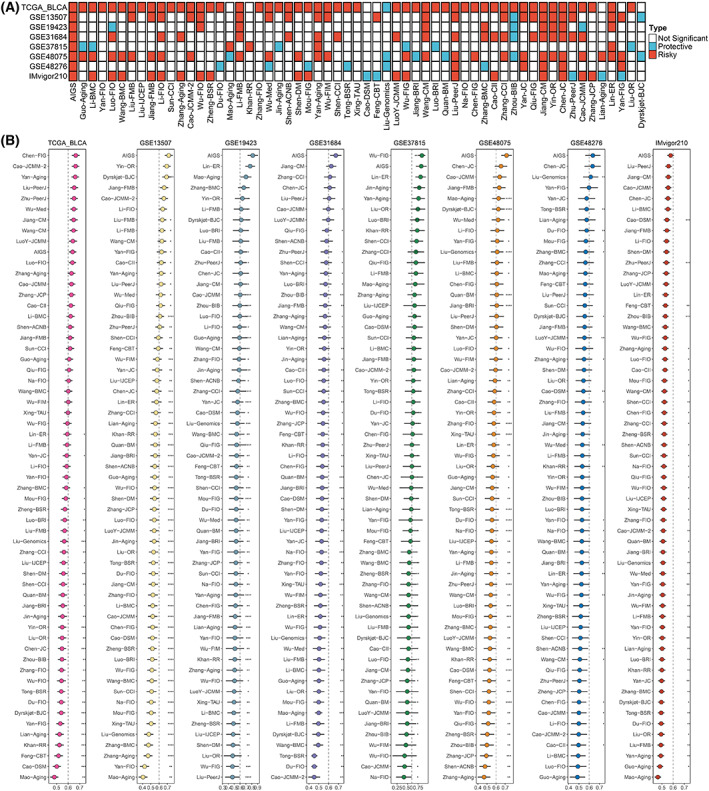
Comparisons between AIGS and gene expression signatures. (A) Univariate cox regression analysis of AIGS and 58 published signatures. (B) C‐indices of AIGS and 58 published signatures in TCGA‐BLCA, GSE13507, GSE19423, GSE31684, GSE37815, GSE48075, GSE48276, and IMvigor210. Z‐score test: *P < 0.05, **P < 0.01, ***P < 0.001, ****P < 0.0001. The error bars indicate 95% confidence interval (CI).

With the rapid development of high‐throughput sequencing and bioinformatics, a large number of prediction models based on gene expression have been reported. To compare whether our AIGS signature is more predictive than other models, a total of 58 mRNA /lncRNA predictive signatures developed by various machine‐learning algorithms, including Lasso, GBM, Ridge, were systematically retrieved (Table S2). Univariate Cox analysis exhibited that AIGS not only remained significant but also displayed superior C‐indices across all eight cohorts (Fig. 5A,B), indicating its robust stability and performance to predict the prognosis risk of BLCA patients. Notably, in the TCGA dataset, a few models with TCGA as the training cohort (for example, Chen, Cao, Yan, Liu, Zhu, etc.) possessed higher C‐indices than AIGS. However, all these signatures performed poorly on other cohorts, suggesting that they suffer from overfitting and frustrating extrapolation. In GSE37815, AIGS ranked second, weaker than Wu, while Wu's performance was relatively poor in other datasets, the C‐indexes in some cohorts were even less than 0.6, which might also be caused by the overfitting.

### 3.5 Nomogram based on AIGS and clinical features

**Fig. 6:**
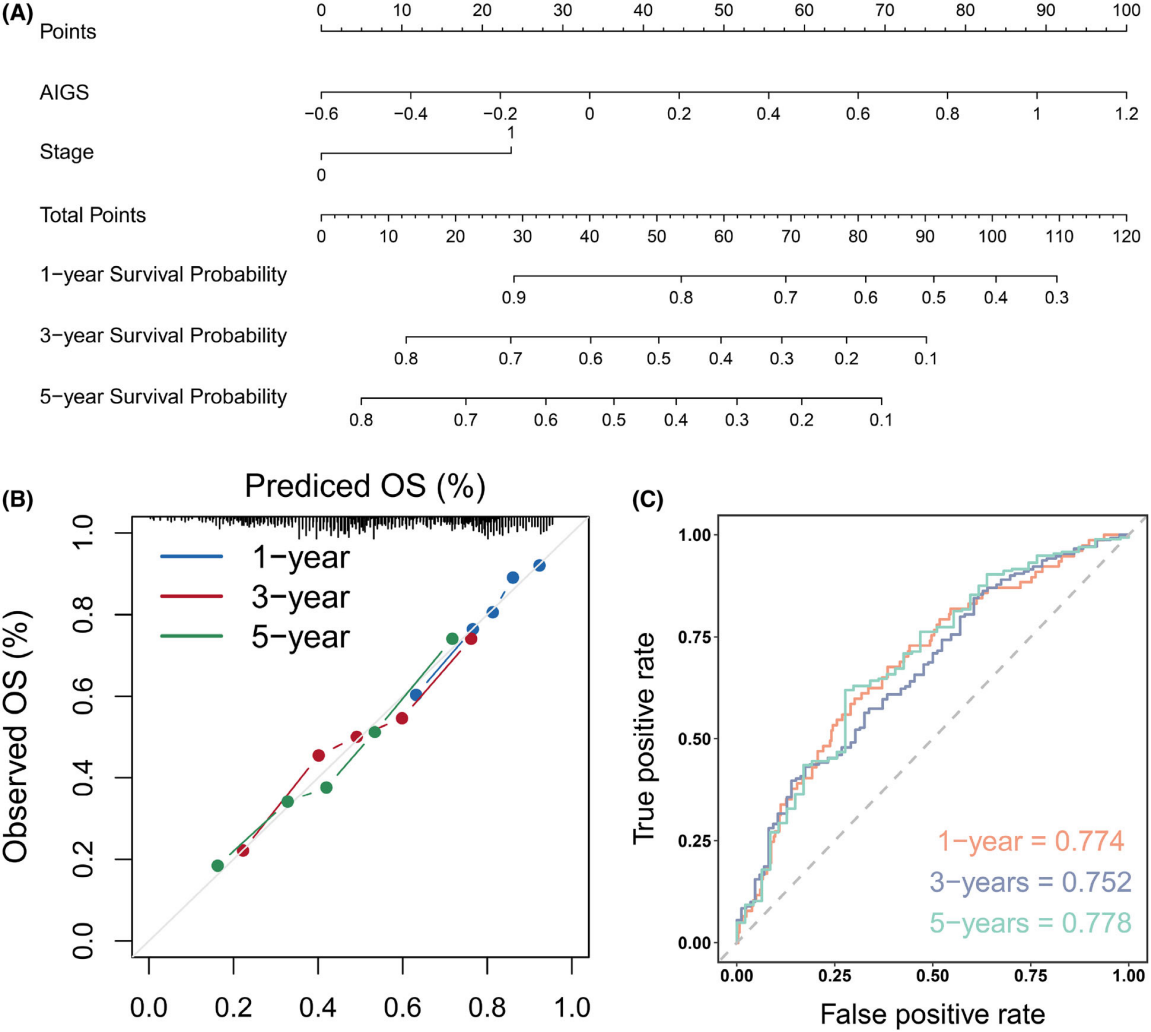
Nomogram construction (A) and performance evaluation (B, C). OS, Overall survival. TCGA‐BLCA (*n* = 400).

Considering the promising clinical application of AIGS, a prognostic nomogram integrated two independent predictors (AIGS and clinical Stage) of mortality was developed (Fig. 6A). Meanwhile, individualized patient scores were calculated to predict the OS of 1, 3, and 5 years. The calibration plot suggested that our nomogram displayed superior performance in predicting the prognosis of BLCA patients (Fig. 6B). Likewise, the AUC values of the nomogram were 0.774/0.752/0.778 at 1/3/5 years (Fig. 6C), indicating its high accuracy and stability.

### 3.6 Biological function analysis of AIGS

To explore the potential functional and molecular mechanisms of AIGS, GSEA and GSVA were subsequently performed. After sorting in descending order according to the absolute value of Normalized Enrichment Score, the top 20 pathways of GO and KEGG were selected, respectively. As displayed in Fig. S1C,D, AIGS‐related genes were mainly enriched in tumour development and metastasis‐related pathways, including DNA replication, chromosome segregation, epidermal cell differentiation, cell cycle, mismatch repair, and ECM receptor interaction. Figure S1E,F revealed that all these pathways were positively correlated with the AIGS score. In parallel, the GSVA result demonstrated that the vast majority of pathways in the Hallmark gene set were significantly different between the high and low‐risk groups, indicating that AIGS was highly correlated with tumours (Fig. S1G).

### 3.7 Low AIGS scores predicted better immune and chemotherapy responses

**Fig. 7:**
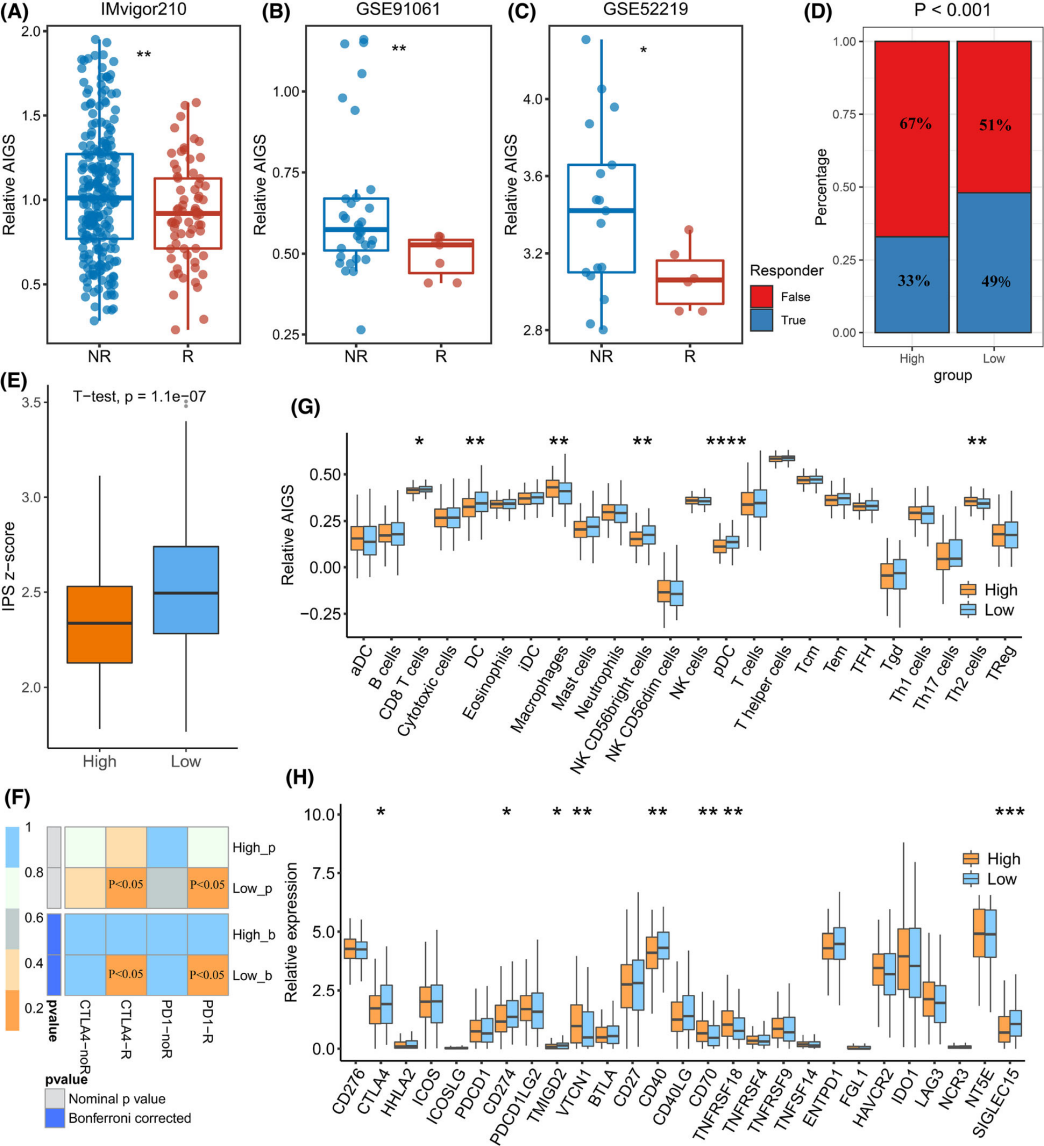
Immunotherapy prediction and immune landscape with regard to AIGS. (A, B) The relationship between AIGS and immunotherapy response in IMvigor210 (*n* = 298) and GSE91061 (*n* = 39); *t*‐test. (C) The relationship between AIGS and chemotherapy response in GSE52219 (*N* = 23); *t*‐test. (D–F) The performance of AIGS in TIDE (D, fisher test), IPS (E, *t*‐test), and SubMap (F) algorithms. (G) The correlation analysis between AIGS and 24 immune cell infiltration abundance; *t*‐test. (H) Boxplot of 27 immune checkpoints profiles between high and low AIGS patients; *t*‐test. The error bars indicate 95% confidence interval (CI). *T*‐test or Wilcoxon rank‐sum test: *P < 0.05, **P < 0.01, ***P < 0.001, ****P < 0.0001. aDC, Activated dendritic cells; AIGS, Artificial intelligence‐derived gene signature; DC, Dendritic cells; iDC, Immature dendritic cells; IPS, Immunophenoscore; pDC, Plasmacytoid dendritic cells; SubMap, Subclass mapping; Tcm, Central memory T cell; Tem, effector memory T cell; TFH, Follicular helper T cell; tgd, T γ δ cells; TIDE, tumour immune dysfunction and exclusion; Treg, regulatory T cells.

To evaluate the predictive potential of AIGS for immunotherapy and chemotherapy, two immunotherapy cohorts (IMvigor210, GSE91061) and one chemotherapy cohort (GSE52219) were retrospectively collected. As illustrated in Fig. 7A,B, a low AIGS score predicted better immune response in both IMvigor210 (*P* < 0.01) and GSE91061(*P* < 0.01) cohorts. The same result was obtained in the chemotherapy cohort (Fig. 7C, *P* < 0.05), revealing that patients with a lower AIGS score respond better to the chemotherapy. Additionally, based on multiple immune response testing algorithms and features like TIDE, IPS, and SubMap, we further verified the predictive performance of AIGS in immune response. The results suggested that patients in the low AIGS group exhibited a higher immune response rate (Fig. 7D), higher IPS score (Fig. 7E), and a superior anti‐PD‐1 and CTLA‐4 immunotherapy efficacy (Fig. 7F), indicating the powerful stability of AIGS in predicting immune response for BLCA.

### 3.8 Immune landscape and potential AIGS immunotherapeutic targets of BLCA

To validate the above results and investigate the underlying mechanism between AIGS and immunity, we explored the immune landscape for BLCA based on immune infiltration and immune checkpoints in the IMvigor210 dataset. As Fig. S2A exhibited, there was a significant difference in immune cell infiltration between the high and low AIGS groups. Notably, the low AIGS group exhibited higher infiltration of CD8 T cells (*P* < 0.05), dendritic cells (*P* < 0.01), NK CD56 bright cells (*P* < 0.01), plasmacytoid dendritic cells (*P* < 0.0001), and lower infiltration of macrophages (*P* < 0.01) and T helper (Th) 2 cells (*P* < 0.01; Fig. 7G). In parallel, the results of immune checkpoint correlation analysis suggested that a low AIGS score predicted higher expression of *CTLA‐4*, *CD274*, *TMIGD2*, *CD40*, and *SIGLEC15* and lower expression of *VTCN1*, *CD70*, and *TNFRSF18* (Figs S2B and 7H), indicating that *CTLA‐4*, *CD274*, *TMIGD2*, *CD40*, and *SIGLEC15* were promising to be the potential targets for immunotherapy.

### 3.9 Somatic mutational landscape, CNVs, and methylation‐driven gene with regard to AIGS

**Fig. 8:**
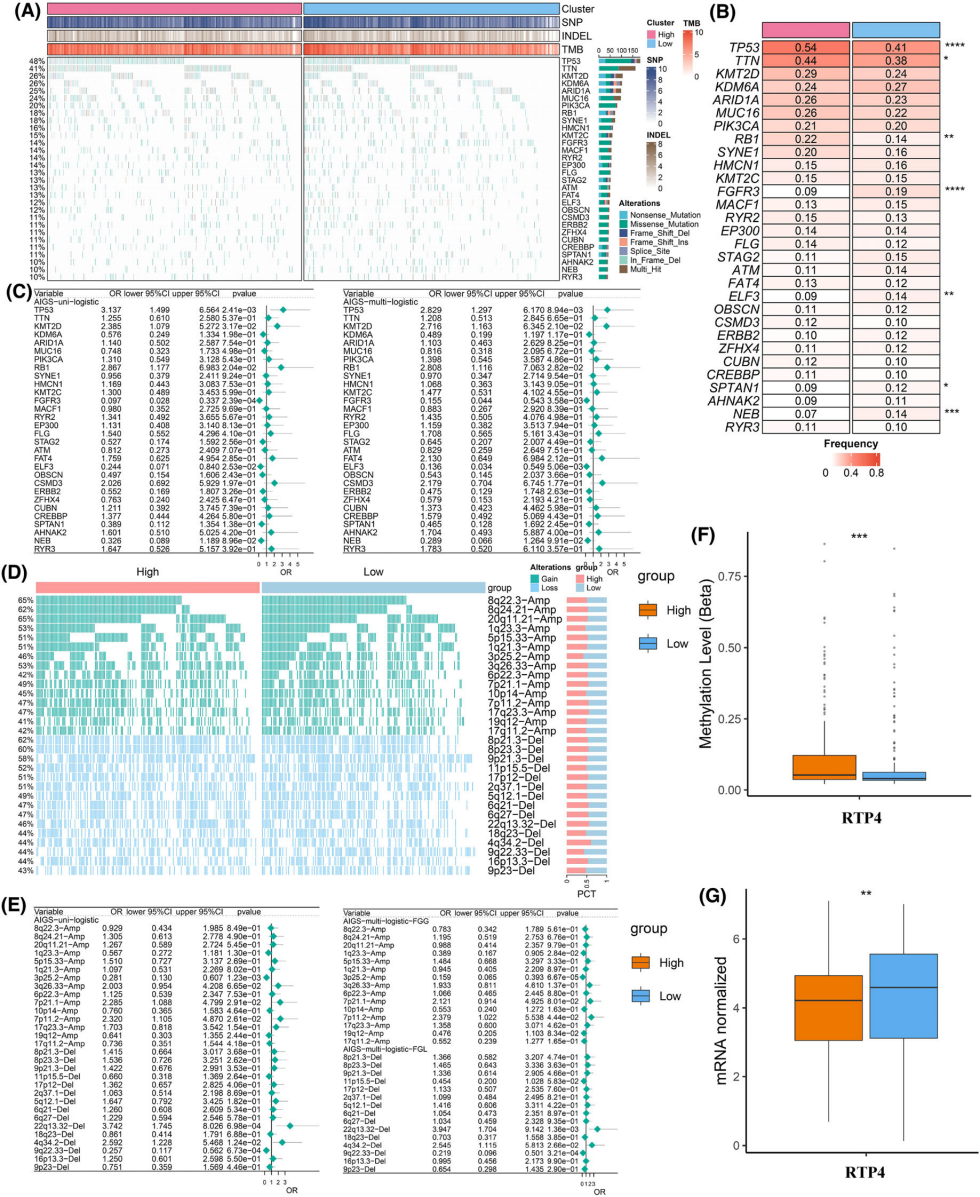
Multi‐omics analysis based on mutation, copy number variations (CNVs) and methylation. (A) The mutational landscape of the top 30 frequently mutated genes (FMGs). (B) The mutation frequency of 30 frequently mutated genes (FMGs) between the high and low AIGS groups; Chisq test. (C) Identification of independent AIGS‐associated mutations by univariate and multivariate logistic regression analysis. (D) The CNV landscape of the top 15 AMP and Homdel chromosome fragments between high and low AIGS patients. (E) Determination of independent AIGS‐related CNV chromosome segment through univariate and multivariate logistic regression analysis. (F, G) Methylation level and expression difference of *RTP4* between high and low AIGS groups; *t*‐test. *P < 0.05, **P < 0.01, ***P < 0.001, ****P < 0.0001. The error bars indicate 95% confidence interval (CI). TCGA‐BLCA (*n* = 400).

The mutational landscape of the top 30 frequently mutated genes (FMGs) was exhibited in Fig. 8A. Overall, seven FMGs exhibited significantly higher mutational frequency between the high and low AIGS groups, including *TP53*, *TTN*, *RB1*, *FGFR3*, *ELF3*, *SPTAN1*, and *NEB* (Fig. 8B). Univariate and multivariate logistic regression analysis demonstrated that AIGS was not only dramatically associated with *TP53*, *RB1*, *FGFR3*, and *ELF3* mutations but also remained an independent significance after adjusting for clinical characteristics such as age, gender, stage, and TMB (Fig. 8C). The CNV status of the top 15 AMP and Homdel chromosome fragments between high and low AIGS groups was further characterized (Fig. 8D). As displayed in Fig. S2C, the amplification of 3p25.2, 3q26.33, 7p21.1, 7p11.2, and deletion of 22q13.32, 4q34.2, and 9q22.33 illustrated significant differences between the high and low AIGS groups. Univariate and multivariate logistic regression suggested that AIGS not only predicted the amplification of 3p25.2, 7p11.2 and the deletion of 22q13.32, 4q34.2, and 9q22.33 but also remained an independent significance after adjusting for clinical characteristics such as age, stage, gender, and FGL/FGG (Fig. 8E).

Through the *MethylMix* r package, 55 MDGs (gene expression was significantly negatively correlated with methylation) were screened. Afterwards, the correlation between these genes and the AIGS score was further calculated via the Wilcox test. As displayed in Fig. S2D, the gene expression and methylation level of *RTP4* were significantly negatively correlated in the high and low AIGS group and all patients. Besides, a higher AIGS score exhibited a higher *RTP4* methylation level (Fig. 8F) and a lower expression (Fig. 8G).

### 3.10 Drug response evaluation and potential therapeutic agents for high‐risk BLCA patients

**Fig. 9:**
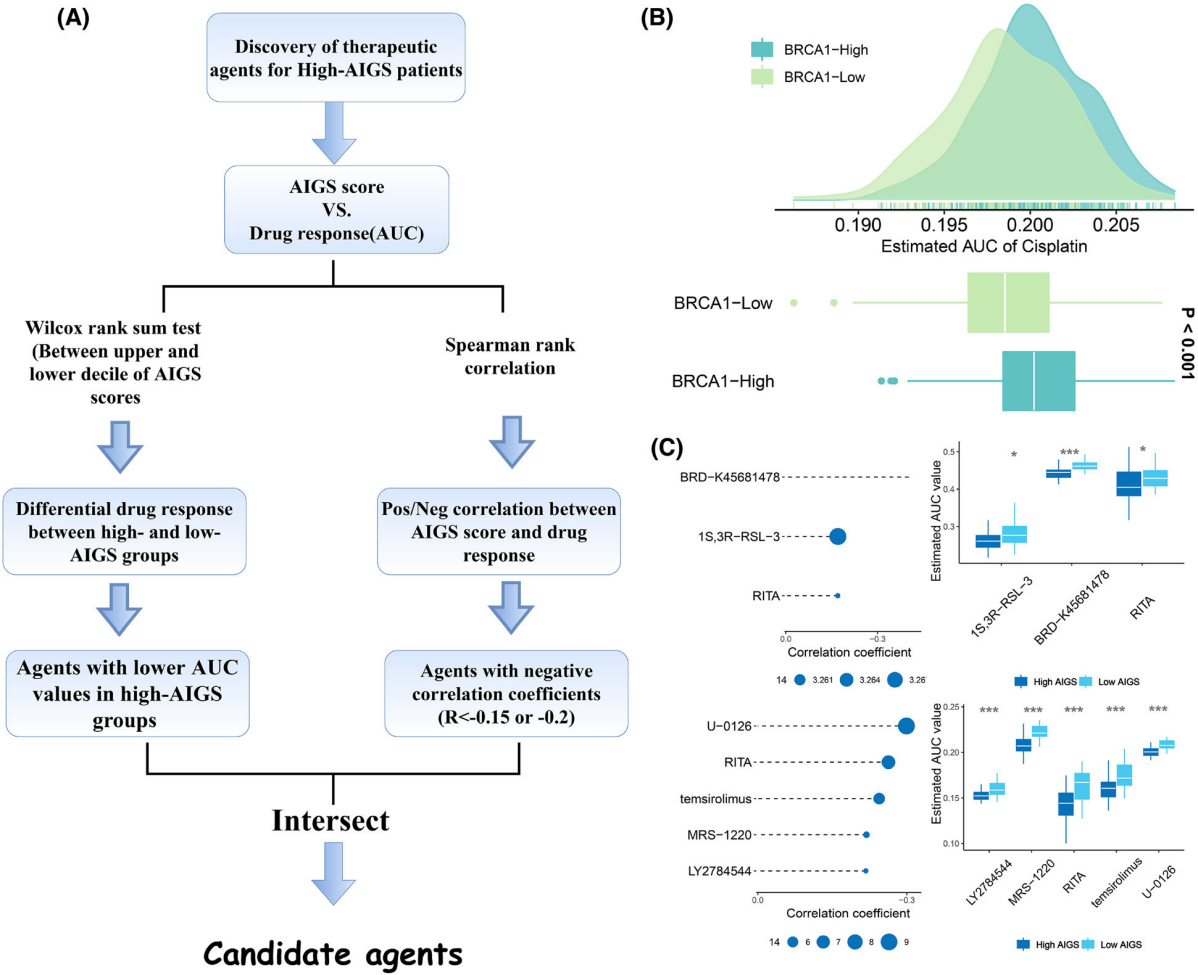
Identification of candidate agents with higher drug sensitivity in high‐AIGS score patients. (A) Schematic outlining the strategy to identify agents with higher drug sensitivity in high AIGS score patients. (B) Comparison of estimated cisplatin's sensitivity between high and low *BRCA1* expression groups; *t*‐test. (C) Potential therapeutic compounds for high‐AIGS BLCA patients; Wilcox test. The error bars indicate 95% confidence interval (CI). *P < 0.05, ***P < 0.001. AIGS, Artificial intelligence‐derived gene signature.

Gene expression and drug sensitivity profiles for hundreds of CCLs are available from the CTRP and PRISM websites to develop predictive models of drug response. After removing compounds with more than 20% of samples with missing values and cell lines from hematopoietic and lymphoid tissues, 266 and 1285 compounds were obtained in the CTRP and PRISM databases, respectively. Afterwards, based on the expression profile, the *pRRophetic* package based on the ridge regression algorism was employed to predict the drug response, resulting in an estimated AUC value for each compound in the sample. To ensure that the obtained drug sensitivities were reliable, cisplatin, a commonly applied drug in neoadjuvant chemotherapy for BLCA was utilized to explore whether the predicted drug sensitivity was consistent with its clinical efficacy. Studies have shown that reduced expression or dysfunction of *BRCA1* predicts a higher cisplatin sensitivity in BLCA [35]. Thus, patients in the TCGA cohort were divided into high‐ and low‐BRCA expression groups based on the median values of *BRCA1* expression. In line with the published research, low *BRCA1* expression patients exhibited a lower cisplatin AUC (*P* < 0.001; Fig. 9B), indicating the remarkable accuracy of estimated drug response.

To obtain potential therapeutic agents for patients with high AIGS scores, two different screening modalities were employed (Fig. 9A). Firstly, differential drug response analysis was performed on patients in the AIGS high and low score groups to select compounds with high AIGS scores and low AUC, followed by Spearman's correlation analysis to screen compounds that displayed significantly negative correlation between AUC values and AIGS (*r* < −0.15 for CTRP or *r* < −0.2 for PRISM). As displayed in Fig. 9C, seven compounds with lower estimated AUC values and higher AIGS scores were finally obtained.

## 4 Discussion

The AJCC classification is a traditional applied clinical management guidance scheme for BLCA. Given the heterogeneity of tumours and the different clinical outcomes of patients with the same stage, AJCC staging displayed significant limitations in prognostic management and risk assessment, which may lead to potential undertreatment or overtreatment [4, 5]. Recently, the rise of immunotherapy has brought new insights into BLCA treatment, significantly extending the OS of patients. However, only a small proportion of patients can benefit from immunotherapy [9]. Thus, the development of stable biomarkers to distinguish the immunotherapy‐sensitive patients accounts for a major challenge in current BLCA immunotherapy [36]. To bridge these gaps, we comprehensively investigated the relationship among gene transcriptome profiles, prognosis, recurrence, and immunotherapy response.

In this study, based on eight independent cohorts, 30 stable OS‐related genes were screened for the development of AIGS. Under the comprehensive application of computer technology, high‐throughput sequencing and bioinformatics, a large number of gene expression prediction models based on various machine‐learning methods have been developed. Notably, why a particular algorithm is employed and which algorithm is optimal deserves further discussion. In fact, the choice of researchers in the algorithm is largely determined by individual preferences and biases. To avoid this problem, we combined 10 machine‐learning algorithms and selected the optimal signature among 76 combined signatures. Eventually, AIGS, a Ridge‐based consensus machine‐learning signature with the highest mean C‐index (0.709) among eight cohorts, was developed, which is also an independent prognostic factor for BLCA prognosis (OS, RFS, PFS). To enable the better clinical application of AIGS, a nomogram was further developed, and it displayed a higher C‐index in the TCGA cohort compared to AIGS alone, indicating a superior predictive value for the prognosis prediction of BLCA.

Some traditional clinical traits (e.g., age, gender, AJCC stage, and smoking) and emerging molecular features (e.g., mutation status of *TP53* and *RB1*) displayed dramatic significance in the prognostic evaluation and clinical management of BLCA, for instance, compared with male, female with BLCA exhibit a worse prognosis and higher risk of death [37]. Patients with *TP53* and *RB1* mutations are generally more aggressive and display worse OS [36]. Thus, we compared the superiority of AIGS with these clinical and molecular features. Across eight independent cohorts, our model not only demonstrated an independent predictive performance after adjusting features like age, gender, AJCC stage, grade, intravesical therapy, systemic chemotherapy, smoking, BCG treatment, platinum therapy, neoadjuvant chemotherapy, *FGFR3*, *p53*, *RAS*, and *RB1* mutations but also presented remarkable superior accuracy in assessing the prognosis risk according to the C‐index assessment, suggesting that it potentially to be a promising surrogate to evaluate the prognosis risk for BLCA in clinical practice.

In addition, we retrospectively collected 58 published signatures consisting of different function genes. The result showed that AIGS maintained relatively superior performance and extrapolation in each cohort compared to these models. Notably, some models performed well in their respective modelling cohorts like Chen, Cao, Yan, Liu, Zhu, et al., but all these signatures performed poor performance in other datasets due to the overfitting.

Based on the above results, AIGS exhibited superior stability in the stratification of high‐ and low‐risk patients; therefore, rational clinical intervention for patients with different levels of AIGS is warranted. Indeed, our AIGS performed high stability in the prediction of immunotherapy response. A lower AIGS predicted higher sensitivity to immunotherapy in both cohorts IMvigor210 and GSE91061, which was further validated in multiple algorithms including TIDE, IPS analysis, and Submap. This finding exhibited far‐reaching implications for the optimization of treatment strategies in patients with BLCA. In addition, lower AIGS also suggested higher chemosensitivity. However, due to the lack of treatment information, this analysis was only performed in one cohort (GSE52219), and the relationship between AIGS and chemotherapy response remains to be confirmed by more clinical and prospective experiments.

Additionally, we explored the underlying biological mechanisms of AIGS. High AIGS was mainly associated with tumour development and metastasis‐related pathways (including DNA replication, chromosome segregation, epidermal cell differentiation, cell cycle, mismatch repair, ECM receptor interaction, etc.). Besides, GSVA results suggested that *MTORC1* signaling and epithelial‐mesenchymal transition pathways were significantly positively correlated with AIGS, which have been reported closely related to tumour cell proliferation, invasion, and metastasis [38, 39]. These results may explain the poor prognosis of patients with high AIGS. Notably, studies have shown that the *E2F* targets pathway is associated with chemotherapy resistance in bladder cancer [40]. Combined with the results of GSVA, patients with low AIGS may be more sensitive to chemotherapy, which paralleled our result in GSE52219.

As we all know, high CD8 T‐cell infiltration generally predicts a better immunotherapy response [41, 42]. Immune cell infiltration results suggested that CD8 T cells exhibited a higher infiltrating abundance in patients with low AIGS, which is consistent with the above results. Over the last decade, ICIs have made a huge splash in cancer treatment by targeting immune checkpoints like *PD‐L1*, *CD40*, and *CTLA‐4* [43]. Our findings performed that the expression of *CTLA‐4*, *CD274* (*PD‐L1*), and *CD40* was significantly increased in patients with low AIGS, which supports our conclusion that low AIGS predicts a better immune response. Besides, the expression of *TMIGD2* and *SIGLEC15* was significantly increased in the low AIGS patients, suggesting that they may be potential immunotherapy targets.

Based on multi‐omics data, we further investigated the mutation and CNV features with regard to AIGS. The result suggested that high AIGS was independently associated with *TP53*, *RB1*, and *ELF3* mutations, and low AIGS was independently related to *FGFR3* mutation (after adjusting for TMB). Studies have performed those mutations of *TP53*, *RB1*, and *ELF3* and revealed the occurrence and poor prognosis of BLCA [44, 45, 46, 47, 48]. Conversely, *FGFR3* mutation predicted a better OS [49]. All these results support the conclusion that high AIGS scores displayed a worse prognosis. Additionally, our study suggested that high AIGS not only predicted the amplification 7p11.2 but also remained an independent significance after adjusting for clinical characteristics such as age, stage, gender, and FGG. Research by Nishiyama N et al. suggested that the amplification of 7p11.2 is tightly related to the development of aggressive non‐papillary BLCA. Consistent with our conclusions, BLCA patients with AIGS may behave more aggressively and display a worse prognosis. Besides, based on the methylation profile, *RTP4*, a novel AIGS‐related methylation driver gene, was identified, which is of great significance for further research considering its tight relationship with AIGS and the gap in this field. Last but not least, based on multiple drug databases as well as comprehensive bioinformatics algorithms, seven compounds with low estimated AUC values and high AIGS scores were finally obtained, which is important for refined treatment of high‐risk BLCA patients.

Although AIGS is a promising comprehensive biomarker, some limitations should be acknowledged. Firstly, all the samples in our study were retrospective, future validation of AIGS should be conducted in prospective fresh samples. Secondly, some clinical and molecular traits on public datasets were very inadequate, which thus had concealed the potential associations between AIGS and some variables. Thirdly, the roles of most genes from AIGS in BLCA remain unknown, and further *in vivo* and *in vitro* experiments are needed to reveal their functions.

## 5 Conclusions

In conclusion, based on multiple bioinformatics and machine‐learning algorithms, we developed a robust and powerful consensus artificial intelligence signature that can accurately predict the prognosis, recurrence, and immune response for BLCA. In addition, AIGS is also a promising biomarker for predicting chemotherapy response, and the identification of potential compounds demonstrates dramatic implications of precise treatment for high‐risk patients. Overall, AIGS is a promising tool to optimize decision‐making and surveillance protocol for individual BLCA patients.

## Conflict of interest

The authors declare no conflict of interest

## Author contributions

ZL and HX contributed study design and data analysis. HX contributed manuscript writing. XH, XG, and JR contributed project oversight and manuscript revisiting. QD, SW, YR, and YZ collected samples and generated data. ZX, SC, and YZ contributed manuscript revisiting.

## Supporting information


**Fig. S1.** Survival and functional analysis of AIGS.
**Fig. S2.** Immune landscape and multi‐omics analysis with regard to AIGS.

**Table S1.** Details of baseline information in 11 public datasets.

**Table S2.** Details of 58 published signatures.

**Table S3.** Annotation information of 30 RORGs.

**Table S4.** Multivariate Cox regression of AIGS regarding to OS.

**Table S5.** Multivariate Cox regression of AIGS regarding to RFS.

**Table S6.** Multivariate Cox regression of AIGS regarding to PFS.

## Data Availability

Public data used in this work can be acquired from the TCGA Research Network portal (https://portal.gdc.cancer.gov/) and Gene Expression Omnibus (GEO, http://www.ncbi.nlm.nih.gov/geo/).

## References

[1] Sung H , Ferlay J , Siegel RL , Laversanne M , Soerjomataram I , Jemal A , et al. Global cancer statistics 2020: GLOBOCAN estimates of incidence and mortality worldwide for 36 cancers in 185 countries. CA Cancer J Clin. 2021;71:209–49. 10.3322/caac.21660 33538338

[2] Antoni S , Ferlay J , Soerjomataram I , Znaor A , Jemal A , Bray F . Bladder cancer incidence and mortality: a global overview and recent trends. Eur Urol. 2017;71:96–108. 10.1016/j.eururo.2016.06.010 27370177

[3] Sell V , Ettala O , Montoya Perez I , Järvinen R , Pekkarinen T , Vaarala M , et al. Symptoms and diagnostic delays in bladder cancer with high risk of recurrence: results from a prospective FinnBladder 9 trial. World J Urol. 2020;38:1001–7. 10.1007/s00345-019-02841-4 31177305 PMC7154016

[4] Martin‐Doyle W , Leow JJ , Orsola A , Chang SL , Bellmunt J . Improving selection criteria for early cystectomy in high‐grade t1 bladder cancer: a meta‐analysis of 15,215 patients. J Clin Oncol. 2015;33:643–50. 10.1200/jco.2014.57.6967 25559810

[5] Rizzo A , Mollica V , Cimadamore A , Santoni M , Scarpelli M , Schiavina R , et al. TNM staging towards a personalized approach in metastatic urothelial carcinoma: what will the future be like?‐a narrative review. Transl Androl Urol. 2021;10:1541–52. 10.21037/tau-20-1109 33850788 PMC8039595

[6] Buchbinder EI , Desai A . CTLA‐4 and PD‐1 pathways: similarities, differences, and implications of their inhibition. Am J Clin Oncol. 2016;39:98–106. 10.1097/coc.0000000000000239 26558876 PMC4892769

[7] Menon S , Shin S , Dy G . Advances in cancer immunotherapy in solid tumors. Cancer. 2016;8:106. 10.3390/cancers8120106 PMC5187504 27886124

[8] Chism DD . Urothelial carcinoma of the bladder and the rise of immunotherapy. J Natl Compr Canc Netw. 2017;15:1277–84. 10.6004/jnccn.2017.7036 28982752

[9] Maiorano BA , De Giorgi U , Ciardiello D , Schinzari G , Cisternino A , Tortora G , et al. Immune‐checkpoint inhibitors in advanced bladder cancer: seize the day. Biomedicine. 2022;10:411. 10.3390/biomedicines10020411 PMC8962271 35203620

[10] Lavallee E , Sfakianos JP , Mulholland DJ . Tumor heterogeneity and consequences for bladder cancer treatment. Cancer. 2021;13:5297. 10.3390/cancers13215297 PMC8582570 34771460

[11] Lu H , Wu J , Liang L , Wang X , Cai H . Identifying a novel defined Pyroptosis‐associated long noncoding RNA signature contributes to predicting prognosis and tumor microenvironment of bladder cancer. Front Immunol. 2022;13:803355. 10.3389/fimmu.2022.803355 35154117 PMC8828980

[12] Wang Z , Zhu L , Li L , Stebbing J , Wang Z , Peng L . Identification of an immune gene‐associated prognostic signature in patients with bladder cancer. Cancer Gene Ther. 2022;29:494–504. 10.1038/s41417-022-00438-5 35169299

[13] Ren W , Zuo S , Yang L , Tu R , Wang P , Zhang X . Identification of a novel immune‐related long noncoding RNA signature to predict the prognosis of bladder cancer. Sci Rep. 2022;12:3444. 10.1038/s41598-022-07286-1 35236887 PMC8891323

[14] Xu F , Tang Q , Wang Y , Wang G , Qian K , Ju L , et al. Development and validation of a six‐gene prognostic signature for bladder cancer. Front Genet. 2021;12:758612. 10.3389/fgene.2021.758612 34938313 PMC8685517

[15] Yerukala Sathipati S , Tsai MJ , Shukla SK , Ho SY , Liu Y , Behesthi A . MicroRNA signature for estimating the survival time in patients with bladder urothelial carcinoma. Sci Rep. 2022;12:4141. 10.1038/s41598-022-08082-7 35264666 PMC8907292

[16] He YH , Deng YS , Peng PX , Wang N , Wang JF , Ding ZS , et al. A novel messenger RNA and long noncoding RNA signature associated with the progression of nonmuscle invasive bladder cancer. J Cell Biochem. 2018. 10.1002/jcb.28089 30426560

[17] Yan M , Jing X , Liu Y , Cui X . Screening and identification of key biomarkers in bladder carcinoma: evidence from bioinformatics analysis. Oncol Lett. 2018;16:3092–100. 10.3892/ol.2018.9002 30127900 PMC6096082

[18] Mariathasan S , Turley SJ , Nickles D , Castiglioni A , Yuen K , Wang Y , et al. TGFβ attenuates tumour response to PD‐L1 blockade by contributing to exclusion of T cells. Nature. 2018;554:544–8. 10.1038/nature25501 29443960 PMC6028240

[19] Liu Z , Guo C , Dang Q , Wang L , Liu L , Weng S , et al. Integrative analysis from multi‐center studies identities a consensus machine learning‐derived lncRNA signature for stage II/III colorectal cancer. EBioMedicine. 2022;75:103750. 10.1016/j.ebiom.2021.103750 34922323 PMC8686027

[20] Liu Z , Liu L , Weng S , Guo C , Dang Q , Xu H , et al. Machine learning‐based integration develops an immune‐derived lncRNA signature for improving outcomes in colorectal cancer. Nat Commun. 2022;13:816. 10.1038/s41467-022-28421-6 35145098 PMC8831564

[21] Subramanian A , Tamayo P , Mootha VK , Mukherjee S , Ebert BL , Gillette MA , et al. Gene set enrichment analysis: a knowledge‐based approach for interpreting genome‐wide expression profiles. Proc Natl Acad Sci USA. 2005;102:15545–50. 10.1073/pnas.0506580102 16199517 PMC1239896

[22] Lambrechts D , Wauters E , Boeckx B , Aibar S , Nittner D , Burton O , et al. Phenotype molding of stromal cells in the lung tumor microenvironment. Nat Med. 2018;24:1277–89. 10.1038/s41591-018-0096-5 29988129

[23] Bindea G , Mlecnik B , Tosolini M , Kirilovsky A , Waldner M , Obenauf AC , et al. Spatiotemporal dynamics of intratumoral immune cells reveal the immune landscape in human cancer. Immunity. 2013;39:782–95. 10.1016/j.immuni.2013.10.003 24138885

[24] Chrétien S , Zerdes I , Bergh J , Matikas A , Foukakis T . Beyond PD‐1/PD‐L1 inhibition: what the future holds for breast cancer immunotherapy. Cancer. 2019;11:628. 10.3390/cancers11050628 PMC6562626 31060337

[25] Janakiram M , Chinai JM , Zhao A , Sparano JA , Zang X . HHLA2 and TMIGD2: new immunotherapeutic targets of the B7 and CD28 families. Onco Targets Ther. 2015;4:e1026534. 10.1080/2162402x.2015.1026534 PMC4570140 26405587

[26] Wang J , Sanmamed MF , Datar I , Su TT , Ji L , Sun J , et al. Fibrinogen‐like protein 1 is a major immune inhibitory ligand of LAG‐3. Cell. 2019;176:334–47.e312. 10.1016/j.cell.2018.11.010 30580966 PMC6365968

[27] Ward‐Kavanagh LK , Lin WW , Šedý JR , Ware CF . The TNF receptor superfamily in co‐stimulating and co‐inhibitory responses. Immunity. 2016;44:1005–19. 10.1016/j.immuni.2016.04.019 27192566 PMC4882112

[28] Charoentong P , Finotello F , Angelova M , Mayer C , Efremova M , Rieder D , et al. Pan‐cancer immunogenomic analyses reveal genotype‐immunophenotype relationships and predictors of response to checkpoint blockade. Cell Rep. 2017;18:248–62. 10.1016/j.celrep.2016.12.019 28052254

[29] Hoshida Y , Brunet JP , Tamayo P , Golub TR , Mesirov JP . Subclass mapping: identifying common subtypes in independent disease data sets. PLoS ONE. 2007;2:e1195. 10.1371/journal.pone.0001195 18030330 PMC2065909

[30] Jiang P , Gu S , Pan D , Fu J , Sahu A , Hu X , et al. Signatures of T cell dysfunction and exclusion predict cancer immunotherapy response. Nat Med. 2018;24:1550–8. 10.1038/s41591-018-0136-1 30127393 PMC6487502

[31] Yang Z , Yan G , Zheng L , Gu W , Liu F , Chen W , et al. YKT6, as a potential predictor of prognosis and immunotherapy response for oral squamous cell carcinoma, is related to cell invasion, metastasis, and CD8+ T cell infiltration. Onco Targets Ther. 2021;10:1938890. 10.1080/2162402x.2021.1938890 PMC8224202 34221701

[32] Mermel CH , Schumacher SE , Hill B , Meyerson ML , Beroukhim R , Getz G . GISTIC2.0 facilitates sensitive and confident localization of the targets of focal somatic copy‐number alteration in human cancers. Genome Biol. 2011;12:R41. 10.1186/gb-2011-12-4-r41 21527027 PMC3218867

[33] Liu Z , Zhang Y , Dang Q , Wu K , Jiao D , Li Z , et al. Genomic alteration characterization in colorectal cancer identifies a prognostic and metastasis biomarker: FAM83A¦IDO1. Front Oncol. 2021;11:632430. 10.3389/fonc.2021.632430 33959500 PMC8093579

[34] Liu Z , Zhang Y , Shi C , Zhou X , Xu K , Jiao D , et al. A novel immune classification reveals distinct immune escape mechanism and genomic alterations: implications for immunotherapy in hepatocellular carcinoma. J Transl Med. 2021;19:5. 10.1186/s12967-020-02697-y 33407585 PMC7789239

[35] Font A , Taron M , Gago JL , Costa C , Sánchez JJ , Carrato C , et al. BRCA1 mRNA expression and outcome to neoadjuvant cisplatin‐based chemotherapy in bladder cancer. Ann Oncol. 2011;22:139–44. 10.1093/annonc/mdq333 20603439

[36] Tran L , Xiao JF , Agarwal N , Duex JE , Theodorescu D . Advances in bladder cancer biology and therapy. Nat Rev Cancer. 2021;21:104–21. 10.1038/s41568-020-00313-1 33268841 PMC10112195

[37] Dobruch J , Daneshmand S , Fisch M , Lotan Y , Noon AP , Resnick MJ , et al. Gender and bladder cancer: a collaborative review of etiology, biology, and outcomes. Eur Urol. 2016;69:300–10. 10.1016/j.eururo.2015.08.037 26346676

[38] Khan FM , Marquardt S , Gupta SK , Knoll S , Schmitz U , Spitschak A , et al. Unraveling a tumor type‐specific regulatory core underlying E2F1‐mediated epithelial‐mesenchymal transition to predict receptor protein signatures. Nat Commun. 2017;8:198. 10.1038/s41467-017-00268-2 28775339 PMC5543083

[39] Li S , Zhou Q , Liu W , Fu Z , Zhao H , Xi S . Targeting SLC1A5 blocks cell proliferation through inhibition of mTORC1 in arsenite‐treated human uroepithelial cells. Toxicol Lett. 2021;345:1–11. 10.1016/j.toxlet.2021.03.007 33781819

[40] Huang CS , Tsai CH , Yu CP , Wu YS , Yee MF , Ho JY , et al. Long noncoding RNA LINC02470 sponges MicroRNA‐143‐3p and enhances SMAD3‐mediated epithelial‐to‐mesenchymal transition to promote the aggressive properties of bladder cancer. Cancer. 2022;14:968. 10.3390/cancers14040968 PMC8870681 35205713

[41] Banchereau R , Chitre AS , Scherl A , Wu TD , Patil NS , de Almeida P , et al. Intratumoral CD103+ CD8+ T cells predict response to PD‐L1 blockade. J Immunother Cancer. 2021;9:e002231. 10.1136/jitc-2020-002231 33827905 PMC8032254

[42] Liu Z , Zhou Q , Wang Z , Zhang H , Zeng H , Huang Q , et al. Intratumoral TIGIT(+) CD8(+) T‐cell infiltration determines poor prognosis and immune evasion in patients with muscle‐invasive bladder cancer. J Immunother Cancer. 2020;8:e000978. 10.1136/jitc-2020-000978 32817209 PMC7430558

[43] Mahoney KM , Rennert PD , Freeman GJ . Combination cancer immunotherapy and new immunomodulatory targets. Nat Rev Drug Discov. 2015;14:561–84. 10.1038/nrd4591 26228759

[44] Brosh R , Rotter V . When mutants gain new powers: news from the mutant p53 field. Nat Rev Cancer. 2009;9:701–13. 10.1038/nrc2693 19693097

[45] Gondkar K , Patel K , Krishnappa S , Patil A , Nair B , Sundaram GM , et al. E74 like ETS transcription factor 3 (ELF3) is a negative regulator of epithelial‐ mesenchymal transition in bladder carcinoma. Cancer Biomark. 2019;25:223–32. 10.3233/cbm-190013 31104013 PMC13082411

[46] Guneri‐Sozeri PY , Erkek‐Ozhan S . Identification of the gene expression changes and gene regulatory aspects in ELF3 mutant bladder cancer. Mol Biol Rep. 2022;49:3135–47. 10.1007/s11033-022-07145-2 35199247

[47] Sjödahl G , Eriksson P , Patschan O , Marzouka NA , Jakobsson L , Bernardo C , et al. Molecular changes during progression from nonmuscle invasive to advanced urothelial carcinoma. Int J Cancer. 2020;146:2636–47. 10.1002/ijc.32737 31609466 PMC7079000

[48] Yan Y , Cai J , Huang Z , Cao X , Tang P , Wang Z , et al. A novel ferroptosis‐related prognostic signature reveals macrophage infiltration and EMT status in bladder cancer. Front Cell Dev Biol. 2021;9:712230. 10.3389/fcell.2021.712230 34490263 PMC8417704

[49] Flippot R , Loriot Y . The FGFR3 story in bladder cancer: another piece of the puzzle? Eur Urol. 2020;78:688–9. 10.1016/j.eururo.2020.08.016 32855010

