# Supplementary material for: Artificial intelligence‐driven consensus gene signatures for improving bladder cancer clinical outcomes identified by multi‐center integration analysis
Source: Mol Oncol. 2022 Sep 22;16(22):4023–42. doi: 10.1002/1878-0261.13313 (PMC9718116; doi:10.1002/1878-0261.13313)
Supplement: Supplementary file 1 — Fig. S1. Survival and functional analysis of AIGS. Fig. S2. Immune landscape and multi‐omics analysis with regard to AIGS. [file MOL2-16-4023-s007.docx]

**
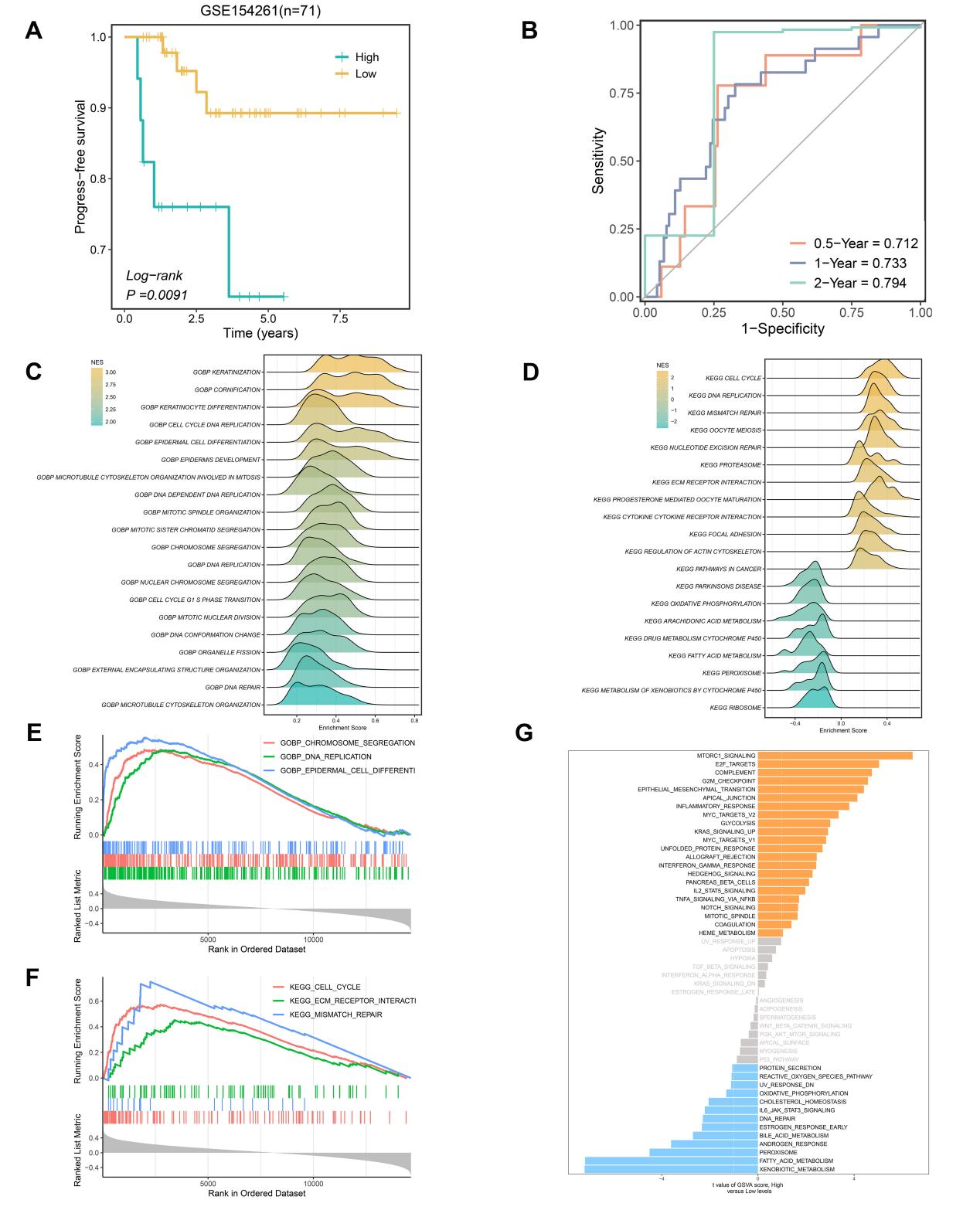
Figure S1. Survival and functional analysis of AIGS. (A)** Kaplan-Meier survival analysis between the high and low AIGS groups in PFS cohort (GSE154261). (**B**) Time-dependent ROC analysis for predicting OS at 0.5, 1, and 2 years in IMvigor210. (**C-D**) The results of GO (**C**) and KEGG (**D**) enrichment analysis by the GSEA algorithm. (**E-F**) The significantly enriched pathways associated with AIGS. (**G**) The result of GSVA between high and low AIGS groups.

**
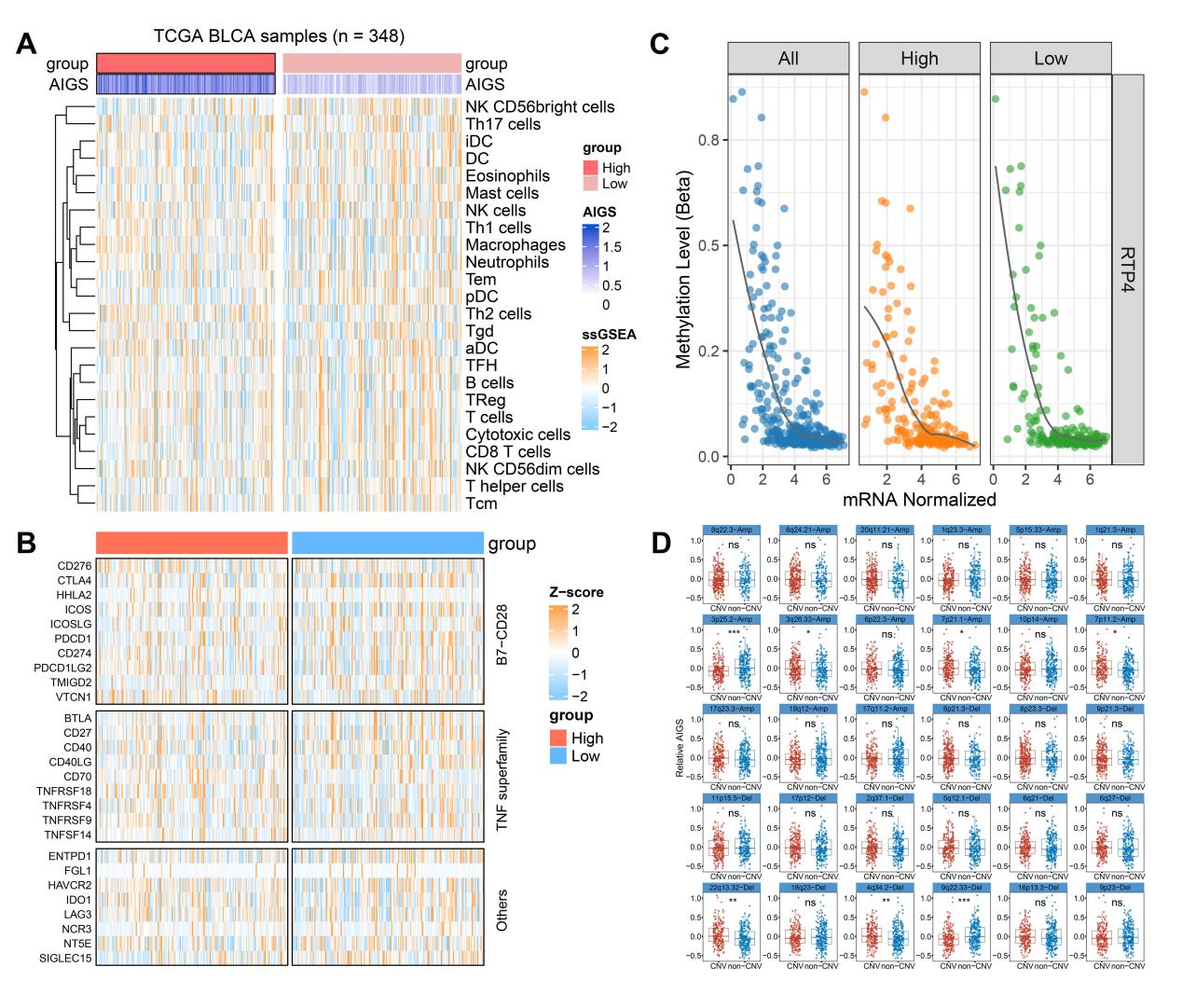
Figure S2. Immune landscape and multi-omics analysis with regard to AIGS.** (**A**) Heatmap of 24 immune cells infiltration abundance between high and low AIGS groups. **(B)** Heatmap of 27 immune checkpoints profiles in high and low AIGS groups. (**C**) Correlation of RTP4 expression and methylation levels in high and low AIGS groups and all patients. (**D**) The CNV difference of the top 15 AMP and Homdel chromosome fragments between two AIGS groups.
